# Study on the Performance of Modified Asphalt Mixture Incorporating MSWI Bottom Ash

**DOI:** 10.3390/ma19132714

**Published:** 2026-06-24

**Authors:** Fanlong Tang, Ting Chen, Yufan Hu, Yinhao Sun

**Affiliations:** 1 College of Network and Communication Engineering, Jinling Institute of Technology, Nanjing 211169, China; 17372262326@163.com; 2 School of Transportation, Southeast University, Nanjing 210096, China; 3 China Railway Construction Urban Construction Transportation Development Co., Ltd., Suzhou 215000, China; s259668@163.com

**Keywords:** pavement engineering, asphalt mixture, MSWI slag, high-temperature stability, water stability, dynamic modulus

## Abstract

To achieve the valorization of municipal solid waste incineration (MSWI) bottom ash and investigate its engineering feasibility as an aggregate replacement in asphalt mixtures, this research adopted MSWI bottom ash in three particle size fractions (2.36–9.5 mm, 9.5–16 mm and 2.36–4.75 mm) to replace basalt aggregate in SBS-modified AC-20 asphalt mixtures. Five dosages of MSWI bottom ash (0%, 7.5%, 15%, 22.5% and 30%) were designed, and high-temperature stability, low-temperature cracking resistance, moisture stability, dynamic modulus and fatigue resistance were tested. The results indicate that the incorporation of MSWI bottom ash causes different degrees of performance degradation. At a dosage of 30%, the dynamic stability of Groups I, II and III decreased by 37.5%, 49.3% and 27.5%, respectively, while the fatigue lives decreased by 48.1%, 60.3% and 31.3%, respectively. The failure strain of Group III at 30% was 2007 microstrain, still slightly higher than the specification limit, whereas Groups I and II dropped to 1825 microstrain and 1575 microstrain. The freeze–thaw splitting tensile strength ratios of Groups I and III at 30% were 81.6% and 84.1%, both meeting the 80% requirement, while Group II decreased to 78.7%. Overall, the 2.36–4.75 mm fraction produced the smallest deterioration, followed by the 2.36–9.5 mm fraction, whereas the 9.5–16 mm fraction showed the most significant reduction. Considering both pavement performance and resource utilization efficiency, MSWI bottom ash is recommended to replace basalt aggregate at dosages not exceeding 30% for the 2.36–4.75 mm fraction and 22.5% for the 2.36–9.5 mm fraction. In addition, the asphalt–aggregate ratio should be adjusted with the slag dosage to compensate for the high absorption of MSWI bottom ash.

## 1. Introduction

The disposal of municipal solid waste is a core aspect of urban solid waste management, with landfill and incineration as the current mainstream treatment methods [1,2,3,4,5]. Although a sanitary landfill is easy to operate and cost-effective, its disadvantages of large land consumption and low resource recycling make it unable to satisfy the demand for urban green and low-carbon development. While the waste incineration process can efficiently degrade hazardous components in municipal solid waste, achieve a volume reduction rate of more than 80%, and concurrently realize resource-oriented utilization for heating and power supply via thermal energy recovery, it has emerged as the preferred technology for MSW treatment in the current phase [6,7,8]. However, waste incineration produces slag accounting for 20–30% of the incinerated waste mass. Taking Lhasa, China, as a case study, the permanent resident population was approximately 876,400 by the end of 2024, and its annual MSWI slag output exceeded approximately 1.2 million tons in 2024. The large-scale stockpiling of such slag not only occupies valuable land resources but also easily triggers secondary environmental pollution problems, including seepage and heavy metal leaching, caused by rainwater leaching. Therefore, realizing the efficient resource utilization of municipal solid waste incineration slag has become a key challenge for improving the urban solid waste treatment industrial chain and promoting the sustainable development of incineration treatment technology.

Researchers worldwide have carried out in-depth investigations into the resource-oriented application of municipal solid waste incineration slag in road engineering [9,10]. These studies primarily examine the mechanical performance, alkali activity, volume expansion properties and leaching characteristics of heavy metals regarding slag utilized as aggregate for road base courses. Studies have demonstrated that MSWI slag possesses the engineering application potential to replace natural aggregate. Vasconcellos et al. [11] evaluated the variability of the modulus of elasticity of concrete caused by different aggregate types and batches, indicating that aggregate source and batch variability can directly affect the mechanical response of cementitious materials. Oliveira et al. [12] assessed the feasibility of using blasted copper slag as artificial fines in ecofriendly concrete, and reported that concrete workability could be enhanced by up to 250% when the slag was used within a controlled dosage range of 0–15%, while the mechanical strength and durability indicators were also improved. These studies suggest that industrial by-product aggregates may be converted into construction materials, but particle characteristics, source variability and incorporation dosage must be carefully controlled. Regarding the resource-oriented utilization of concrete, Fang [13] summarized the research advances of MSWI slag in concrete. They systematically investigated the fundamental physicochemical characteristics of slag and concluded that its application in concrete must balance mechanical properties and environmental hazards via the optimization of pretreatment techniques and incorporation ratios. Prospective studies are recommended to concentrate on the exploitation of composite blends, the assessment of concrete long-term durability, and the reinforcement of sustained monitoring for heavy metal leaching. In view of the whole industrial chain of resource-oriented utilization, Xu [14] concluded that municipal solid waste incineration slag is chemically stable, owing to high-temperature incineration. Enriched with recyclable metals and cementitious constituents, the slag provides the prerequisite for versatile resource utilization applications, thereby qualifying as a solid waste resource that integrates environmental and economic advantages. In terms of road base applications, Zhou [15] centered on the engineering utilization of incineration slag bottom ash in road base layers, and investigated its physical properties, chemical constituents and heavy metal leaching behavior by means of experimental testing. The findings demonstrate that MSWI slag bottom ash presents comparable physical and chemical characteristics to natural aggregate, laying a feasible foundation for its utilization in road base construction. Furthermore, the incorporation ratio of bottom ash has a twofold impact on the mechanical performance of road bases, which necessitates the optimization of material proportions to realize effective performance control. Aiming at the hydration and carbonation properties of slag from a microscopic perspective, Dong [16] evaluated the pavement performance of asphalt mixtures blended with recycled aggregate and incineration slag aggregate. On the basis of mix design optimization and laboratory performance evaluations, the results revealed that an appropriate incorporation ratio could synergistically enhance the mixture performance, and the optimal gradation scheme for overall performance was subsequently identified. Building upon this foundation, Zhang [17] explored the influence of titanium-bearing blast furnace slag on the microstructure and hydration properties of belite cement. The experimental results verified that an appropriate dosage of blast furnace slag is capable of optimizing the clinker phase constitution and remarkably enhancing the compressive strength of cement at different curing ages. It can be summarized from the aforementioned studies that utilizing slag as road paving materials is technically feasible, and such application has been implemented in engineering practice of cement concrete construction. When applied to asphalt pavements as a raw material, the particle size and incorporation ratio of slag significantly affect the pavement performance of asphalt mixtures. Nevertheless, the detailed regulation and mechanism of its blending proportion warrant further in-depth investigation.

Accordingly, this paper takes SBS-modified asphalt mixture, a commonly used material for the middle course of road pavements, as the research object. Municipal solid waste incineration slag is adopted to replace basalt coarse aggregate in three different particle size ranges. Mixture specimens are prepared with gradient dosages of slag set from 0% to 30%. The high-temperature stability, low-temperature crack resistance, water stability, dynamic modulus and fatigue resistance of the mixtures are systematically tested. The influence laws of MSWI slag with different particle sizes and dosages on the pavement performance of asphalt mixtures are clarified. The optimal particle size range and dosage range of MSWI slag applied in SBS-modified asphalt mixture are determined. This research is intended to provide an experimental basis and technical reference for the large-scale and standardized resource utilization of MSWI slag in asphalt pavement engineering. From the perspective of the United Nations Sustainable Development Goals, this work is related to SDG 9 (Industry, Innovation and Infrastructure), SDG 11 (Sustainable Cities and Communities), SDG 12 (Responsible Consumption and Production), and SDG 13 (Climate Action), because it aims to reduce natural aggregate consumption and promote the high-value recycling of municipal solid waste.

## 2. Materials and Methods

### 2.1. Municipal Solid Waste Incineration Slag

MSWI slag utilized in the experiments was sourced from an urban municipal solid waste incineration plant [18,19]. After natural cooling and manual sorting to eliminate impurities such as metals and plastics, the slag was crushed and sieved to the particle size ranges specified for the tests. The main chemical components of MSWI slag include SiO_2_, CaO, Fe_2_O_3_ and Al_2_O_3_, whose specific contents are presented in Table 1. Its inorganic mineral composition is analogous to that of natural aggregates, which meets the basic criteria for serving as an alternative aggregate material. The physical and mechanical indicators of the aggregates and mineral fillers, together with the corresponding test standards, are compared in Table 2.

In accordance with the technical requirements for aggregates stipulated in Technical Specifications for Construction of Highway Asphalt Pavements JTG F40-2017 [21], the key performance indicators of MSWI slag were tested and compared with those of basalt coarse aggregate, limestone fine aggregate and mineral filler. As shown in Table 2, the crushing value, high-temperature crushing value, Los Angeles abrasion loss, flaky and elongated particle content, and water absorption of MSWI bottom ash are all much higher than those of basalt aggregate. The Los Angeles abrasion loss of basalt aggregate was rechecked as the average value of parallel tests and confirmed to be 8.2%. The content of particles smaller than 0.075 mm tested by the water washing method is also relatively high, and its apparent density and bulk specific gravity are lower than those of basalt aggregate. This indicates that the mechanical and physical properties of MSWI slag aggregate are poor, so it is not suitable to directly replace large-size basalt aggregate with MSWI slag in a large proportion.

### 2.2. Asphalt and Other Aggregates

SBS-modified asphalt was adopted in the experiment. Its various performance indicators were tested in accordance with Standard Test Methods of Bitumen and Bituminous Mixtures for Highway Engineering JTG E20-2011 [22], and the test results are listed in Table 3. The corresponding test methods include penetration (T0604), ductility (T0605), softening point (T0606), viscosity (T0625), density (T0603) and RTFOT aging indexes (T0610). All indicators meet the technical requirements for modified asphalt specified in JTG F40-2017. Basalt was adopted as the coarse aggregate, limestone as the fine aggregate, and limestone powder as the mineral filler. The performance indicators of the three materials are listed in Table 2, all of which comply with the specification’s technical requirements for aggregates used in asphalt mixtures, serving as the benchmark aggregates for the experiment [23,24].

### 2.3. Design and Preparation of Asphalt Mixtures

The experiment adopted the gradation of AC-20 asphalt mixture. The sieve passing rates of this gradation are presented in Table 4, and the gradation curve conforms to the recommended range specified in JTG F40-2017. Based on the performance characteristics of MSWI slag [25], three experimental groups with different blending intervals were designed, and the specific divisions are as follows.

Group I: MSWI slag was adopted to replace basalt aggregate with a particle size of 2.36–9.5 mm exclusively, and the aggregate in this grading interval accounted for 33.6% of the total mass of aggregates in the mixture.

Group II: MSWI slag was adopted to replace basalt aggregate with a particle size of 9.5–16 mm exclusively, and the aggregate in this grading interval accounted for 34% of the total mass of aggregates in the mixture.

Group III: MSWI slag was adopted to replace basalt aggregate with a particle size of 2.36–4.75 mm exclusively, and the aggregate in this grading interval accounted for 10.2% of the total mass of aggregates in the mixture.

Each group was designed with five dosages of MSWI slag: 0, 7.5%, 15%, 22.5% and 30%. The dosage refers to the percentage of the mass of aggregate in the corresponding particle size interval. The optimum asphalt–aggregate ratio (OAC) of the mixture at each slag dosage was determined through the Marshall Test. When water accumulates on the asphalt pavement, the asphalt binder may experience stripping and accelerated aging under the combined action of moisture, repeated traffic loading, oxygen and ultraviolet radiation, thereby triggering moisture damage to the asphalt pavement. To investigate the water stability of asphalt mixtures incorporating MSWI slag at various dosages, freeze–thaw splitting cycle tests were carried out in accordance with JTG E20-2011 on both plain asphalt mixtures and SBS-modified asphalt mixtures with different slag contents. The water stability of asphalt mixtures at different slag dosages was evaluated based on the tensile strength ratio (TSR) [26].

Due to the high water absorption characteristic of MSWI slag, the asphalt–aggregate ratio of the mixture was appropriately increased to ensure the thickness of the asphalt film on the aggregate surface, with reference to the design experience of asphalt mixtures incorporating steel slag aggregate [27,28,29,30], as shown in Table 4. The characteristics of the experimental series are summarized in Table 5. In accordance with the provisions of Standard Test Methods of Bitumen and Bituminous Mixtures for Highway Engineering (JTG E20-2011), the mixture specimens of each experimental group were prepared using the gyratory compaction method for subsequent pavement performance tests, and 100 gyrations were adopted for all laboratory specimens.

### 2.4. Performance Tests of Asphalt Mixtures

In accordance with Standard Test Methods of Bitumen and Bituminous Mixtures for Highway Engineering JTG E20-2011 and Technical Specifications for Construction of Highway Asphalt Pavements JTG F40-2017, combined with the existing performance test methods for asphalt mixtures incorporating MSWI bottom ash, pavement performance tests were carried out on the mixtures of each experimental group [31,32,33]. Unless otherwise specified, no fewer than three parallel specimens were tested for each index, and the mean values were used for comparative analysis. The specific test items and test bases are listed as follows:

(1) High-Temperature Stability: The rutting test (T0719) was conducted in accordance with JTG E20-2011 to measure the dynamic stability (DS) of the mixtures under the conditions of 60 °C and 0.7 MPa. The wheel-tracking loading frequency was 42 passes/min, the test duration was 60 min, and DS was calculated from the deformation difference between 45 min and 60 min, corresponding to approximately 630 passes in the calculation interval.
(2) Low-Temperature Crack Resistance: The low-temperature bending test (T0715) was adopted to test the failure strain of the mixtures at −10 °C, so as to evaluate its low-temperature cracking resistance.
(3) Moisture Stability: The freeze–thaw splitting test (T0729) was performed to test the splitting tensile strength of the mixtures before and after freeze–thaw cycles. The tensile strength ratio (TSR) was calculated to evaluate its moisture damage resistance.
(4) Dynamic Modulus: The uniaxial compression dynamic modulus test was carried out in accordance with JTG E20-2011/T0738 at 20 °C with six loading frequencies (0.1, 0.5, 1, 5, 10 and 25 Hz) to measure the dynamic modulus of the mixtures, so as to evaluate their viscoelastic response and mechanical bearing capacity.
(5) Fatigue Resistance: The four-point bending fatigue test (T0739) was implemented to test the fatigue life of the mixtures at 15 °C with a loading frequency of 10 Hz, so as to evaluate its fatigue failure resistance.

## 3. Results and Discussion

### 3.1. High-Temperature Stability

**Figure 1:**
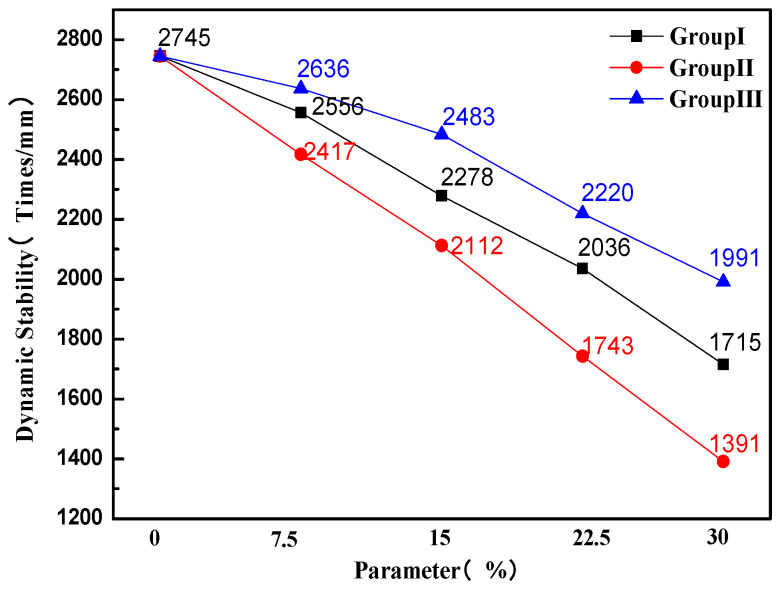
Dynamic stability (cycles/mm) of AC20 asphalt mixtures with various MSWI slag dosages in different blending groups.

The test results of dynamic stability for each experimental group at various MSWI slag dosages are presented in Figure 1. It can be seen from Figure 1 that within the same blending interval, as the dosage of MSWI slag increases from 0 to 30%, the dynamic stability of the mixture exhibits a continuous downward trend, indicating that the incorporation of slag significantly deteriorates the high-temperature rutting resistance of the mixture. At the same MSWI slag dosage, the dynamic stability of the three groups of mixtures ranks as Group III > Group I > Group II. This indicates that the smaller the particle size of the incorporated slag is, the better the high-temperature stability of the mixture will be. Group II, where slag is blended to replace the aggregate in the large particle size range of 9.5–16 mm, shows the most significant performance degradation, which is consistent with the conclusions of existing research [8].

When the MSWI slag dosage reaches 30%, the dynamic stability of Group I, Group II and Group III decreases by 37.5%, 49.3% and 27.5% respectively compared with the control group (0% dosage). In accordance with the specification requirements of JTG F40-2017, the dynamic stability of modified asphalt mixtures should be no less than 2000 times/mm for pavements in hot summer regions. The dynamic stability of Group I at 22.5% slag dosage and Group III at 30% slag dosage still meets the specification requirements, whereas that of Group II falls below the specification limit at a slag dosage of 15%.

The reasons for the degradation of the mixture’s high-temperature stability induced by MSWI slag are as follows: Firstly, MSWI slag itself features a high crushing value and abrasion loss with low mechanical strength as an aggregate, making it prone to crushing and deformation under the combined action of high temperature and traffic loads. Meanwhile, MSWI slag exhibits a high water absorption capacity and adsorbs more asphalt during the mixing process, which reduces the thickness of the effective asphalt film between aggregates and weakens the inter-aggregate cohesion. Lastly, large-sized MSWI slag aggregates feature a high content of needle-flaky particles, leading to an insufficiently compact interlocking structure of aggregates inside the mixture, which is prone to plastic deformation at high temperatures. Conversely, the content of needle-flaky particles in MSWI slag aggregates within the small particle size range (2.36–4.75 mm) is relatively low. Moreover, these aggregates function as fillers in the mixture and impose a slight impact on the aggregate interlocking structure, thus leading to the minimal degradation extent of high-temperature stability.

### 3.2. Low-Temperature Crack Resistance

**Figure 2:**
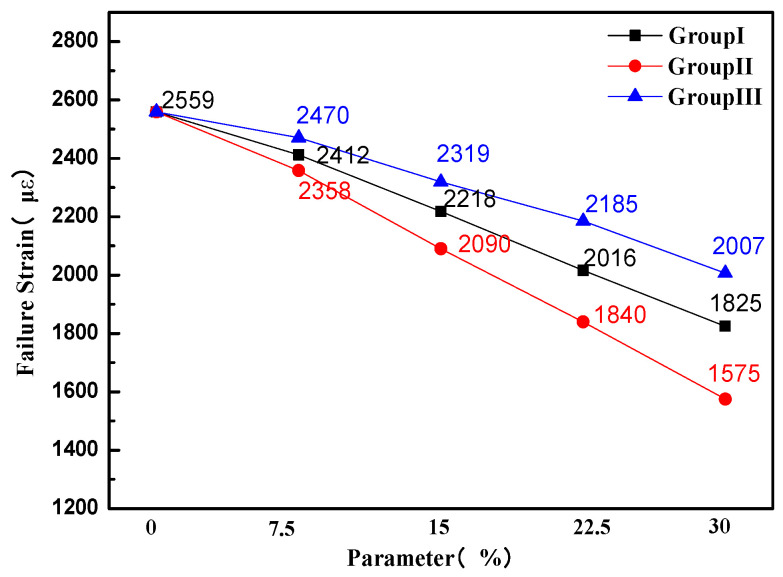
Failure strain (με) of AC20 asphalt mixtures with various MSWI slag dosages in different blending groups.

The test results of failure strain for AC20 asphalt mixtures with various MSWI slag dosages in each experimental group are presented in Figure 2. It can be seen from Figure 2 that the variation law of the mixture’s low-temperature failure strain is consistent with that of its high-temperature dynamic stability [34,35]. Within the same blending interval, the higher the MSWI slag dosage, the lower the failure strain and the poorer the low-temperature crack resistance. Furthermore, the results indicate that the incorporation of small-sized MSWI slag exerts a slighter impact on the low-temperature crack resistance of the mixture [36]. This is attributed to the fact that small-sized MSWI slag possesses a larger contact area with asphalt, resulting in more sufficient interfacial bonding. Meanwhile, it imposes a weaker constraint on the overall deformation of the mixture, allowing the material to better accommodate low-temperature shrinkage deformation.

The failure strain of the mixture in the control group (0% dosage) is 2559 με, which satisfies the requirement in JTG F40-2017 that the low-temperature failure strain of modified asphalt mixtures should be no less than 2000 με. When the MSWI slag dosage reaches 30%, the failure strain of Group III is 2007 με, still slightly higher than the specification limit, while the values of Group I and Group II are 1825 με and 1575 με respectively, both falling below the specification limit. The primary reason for the degradation of the mixture’s low-temperature crack resistance with the increase in MSWI slag dosage lies in the poor adhesion between MSWI slag aggregates and asphalt. Microcracks tend to initiate at the interfacial bonding zone under low-temperature conditions and propagate rapidly. Furthermore, MSWI slag itself features high brittleness and inferior deformation resistance, rendering it prone to cracking under the effect of low-temperature shrinkage stress.

### 3.3. Water Stability

**Figure 3:**
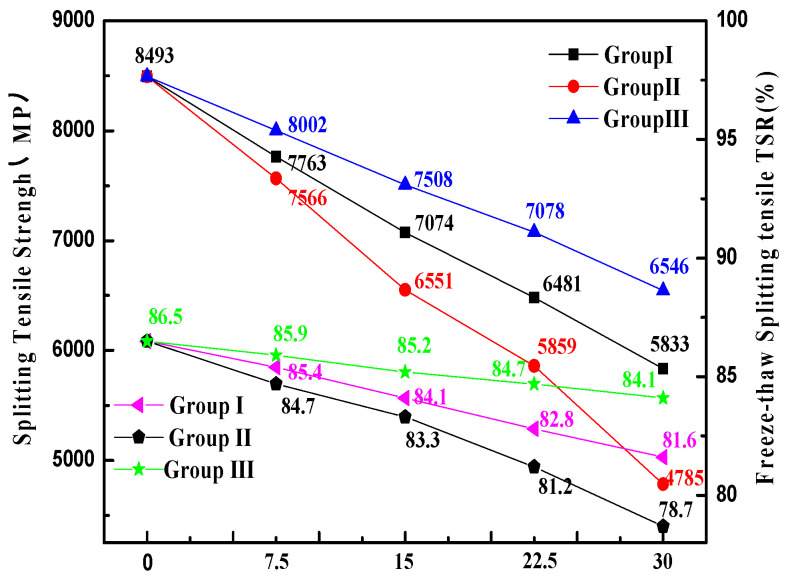
Freeze–thaw splitting tensile strength ratio (TSR, %) of AC20 asphalt mixtures with various MSWI slag dosages in different blending groups.

The test results of the freeze–thaw splitting tensile strength ratio (TSR) for AC20 asphalt mixtures with various MSWI slag dosages in each experimental group are presented in Figure 3. It can be observed from Figure 3 that with the increase in MSWI slag dosage, the freeze–thaw splitting tensile strength ratios (TSRs) of the three groups of mixtures all exhibit a gradual downward trend, indicating a continuous degradation in water stability [37]. At the same MSWI slag dosage, the water stability of the mixtures is ranked as Group III > Group I > Group II, which is consistent with the variation laws of their high- and low-temperature performance.

The freeze–thaw splitting tensile strength ratio (TSR) of the mixture in the control group is 86.5%, which meets the technical requirement in specification JTG F40-2017 that the TSR value of modified asphalt mixtures should be no less than 80%. When the MSWI slag dosage increases to 30%, Group I (81.6%) and Group III (84.1%) still meet the specification requirements, whereas Group II (78.7%) falls below the limit. Even at an MSWI slag dosage of 22.5%, the freeze–thaw splitting tensile strength ratio (TSR) of Group II (81.2%) is close to the lower specification limit.

The reasons why MSWI slag reduces the water stability of the mixture are as follows: First, MSWI slag features a high water absorption capacity. During freeze–thaw cycles, the water absorbed inside the aggregates freezes and expands, generating internal stress that leads to bonding failure at the aggregate–asphalt interface. Second, MSWI slag possesses a highly hydrophilic surface and is rich in SiO_2_ and metal oxides, leading to a low adhesion grade with asphalt. Moisture readily intrudes into the interfacial zone, damaging the bonding between the asphalt film and aggregates and consequently inducing moisture damage. Third, large-sized MSWI slag aggregates have a high void content, allowing moisture to penetrate more easily into the interior of the mixture and thus exacerbating freeze–thaw damage. In contrast, the mixture incorporated with small-sized MSWI slag in Group III features a denser internal void structure, which makes it difficult for moisture to intrude, thus resulting in the minimal degradation extent of water stability.

### 3.4. Dynamic Modulus

**Figure 4:**
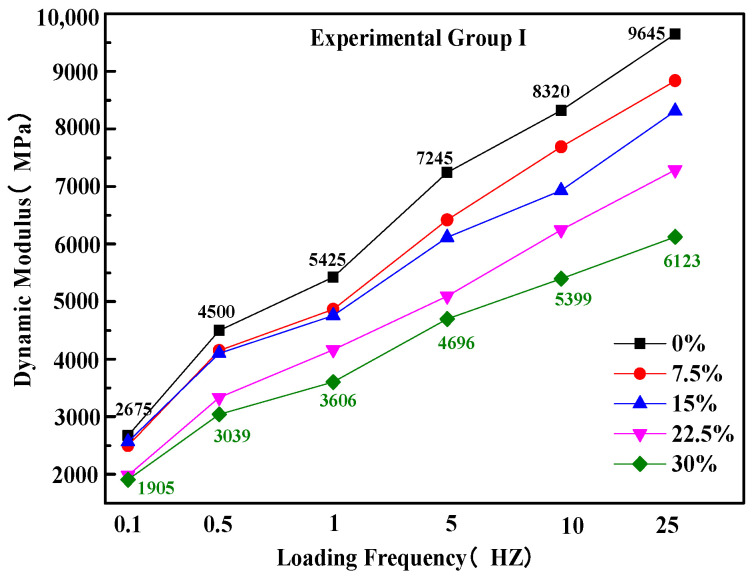
Dynamic modulus (MPa) of AC20 mixtures with various MSWI slag dosages for Group I (2.36–9.5 mm).

**Figure 5:**
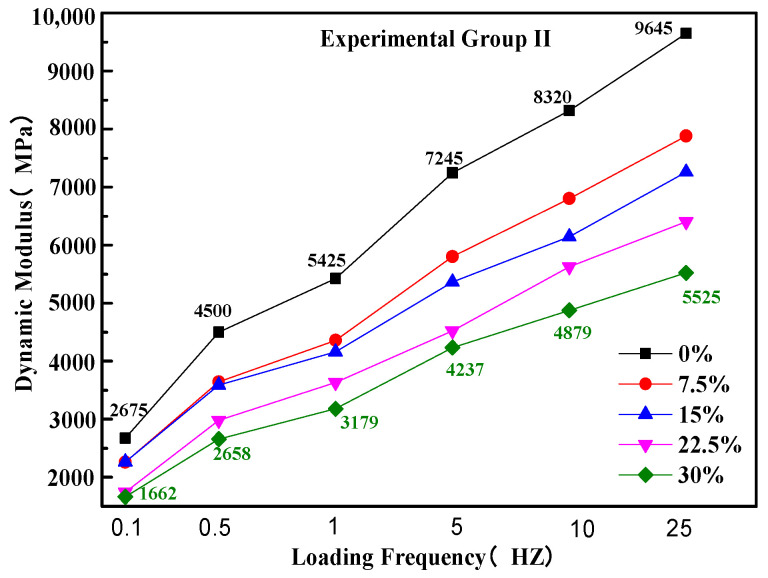
Dynamic modulus (MPa) of AC20 mixtures with various MSWI slag dosages for Group II (9.5–16 mm).

**Figure 6:**
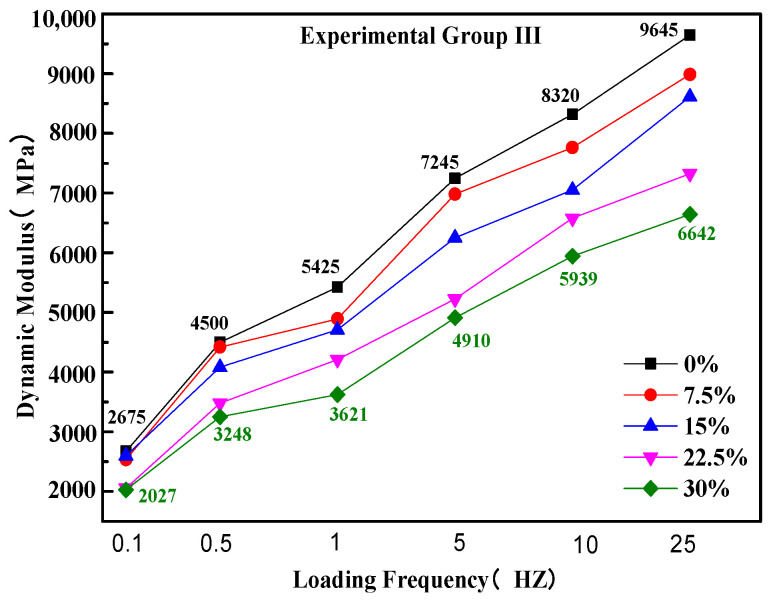
Dynamic modulus (MPa) of AC20 mixtures with various MSWI slag dosages for Group III (2.36–4.75 mm).

At 20 °C, the test results of the dynamic modulus for AC20 asphalt mixtures with various MSWI slag dosages (0, 7.5%, 15%, 22.5%, 30%) under different loading frequencies in each experimental group are presented in Figure 4, Figure 5 and Figure 6. Accordingly, the dynamic modulus results were further discussed from two aspects, namely loading frequency and MSWI slag dosage. Dynamic modulus is a critical indicator for evaluating the viscoelasticity and mechanical bearing capacity of asphalt mixtures, and its variation exhibits three major laws:

(1) Frequency Loading Effect: Within the same experimental group and at the same MSWI slag dosage, as the loading frequency increases from 0.1 Hz to 25 Hz, the dynamic modulus of the mixture presents a significant upward trend, demonstrating distinct viscoelastic characteristics. The mixture behaves elastically with strong bearing capacity at high frequencies, whereas it exhibits viscous behavior with weak bearing capacity at low frequencies.
(2) Dosage Effect: Within the same experimental group and at the same loading frequency, the dynamic modulus of the mixture decreases continuously with the increase in MSWI slag dosage. The higher the slag dosage, the poorer the mechanical bearing capacity.
(3) Blending Interval Effect: At the same MSWI slag dosage and loading frequency, the dynamic modulus of the mixtures is ranked as Group III > Group I > Group II. The incorporation of small-sized MSWI slag exerts the minimal negative impact on the mechanical properties of the mixture [38,39].

Taking the commonly used conditions of 20 °C and 10 Hz loading frequency in pavement structure design as an example, at an MSWI slag dosage of 20%, the dynamic moduli of Group I, Group II and Group III decrease by 35.1%, 41.4% and 28.6% respectively, compared with the control group (0% dosage), indicating that the mixture incorporated with small-sized MSWI slag still maintains favorable mechanical bearing capacity. The main reason for the decrease in the dynamic modulus of the mixture due to the incorporation of MSWI slag is that the strength and stiffness of MSWI slag aggregates are lower than those of basalt aggregates. Furthermore, the internal interlocking structure and bonding performance of the mixture degrade, making the mixture prone to deformation under external forces and weakening its mechanical response, which is consistent with the findings of Joumblat et al. that MSWI-derived fly ash can affect the dynamic modulus and phase angle responses of asphalt concrete mixtures [40].

### 3.5. Fatigue Resistance

**Figure 7:**
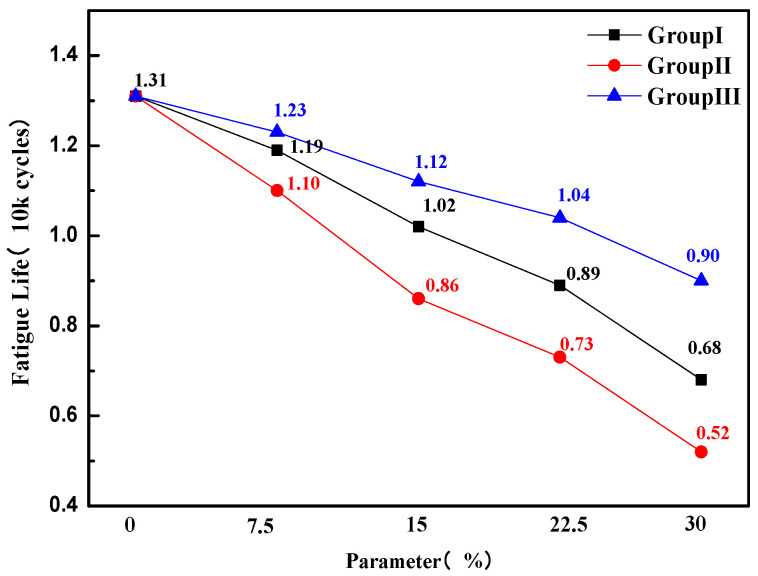
Fatigue life (10^4^ cycles) of AC20 mixtures with various MSWI slag dosages in different blending groups.

The four-point bending fatigue life test results of AC20 asphalt mixtures with various MSWI slag dosages in each experimental group are presented in Figure 7. It can be seen from Figure 7 that the variation law of the fatigue life of the mixture is highly consistent with the aforementioned pavement performance: within the same blending interval, the higher the MSWI slag dosage, the shorter the fatigue life and the worse the fatigue resistance; at the same dosage, the fatigue life is ranked as Group III > Group I > Group II, and the mixture incorporated with small-sized slag in the 2.36~4.75 mm particle size range presents the strongest resistance to fatigue failure.

The fatigue life of the mixture in the control group is 13,100 cycles. At an MSWI slag dosage of 30%, the fatigue life of Group III is 9000 cycles, representing a decrease of 31.3% compared with the control group, while the fatigue lives of Group I and Group II decrease by 48.1% and 60.3% respectively. Analysis reveals that the bonding interface between MSWI slag and asphalt tends to be the initiation site of fatigue cracks, which is the main reason for the degradation of the mixture’s fatigue resistance [41]. Meanwhile, under repeated loading, the microcracks at the interface propagate rapidly and interconnect with each other. Furthermore, MSWI slag aggregates themselves exhibit inferior mechanical properties and are susceptible to fatigue fracture under cyclic loading, which ultimately results in the structural failure of the entire mixture. In Group III, the small-sized MSWI slag aggregates possess a larger bonding area with asphalt, and the interfacial stress is distributed more uniformly. Accordingly, the test results demonstrate that the initiation and propagation of fatigue cracks occur at a slower rate, endowing the mixture with superior fatigue resistance.

## 4. Case Project for Discussion

### 4.1. Case Project Overview

The test sections are located on Provincial Highway named S303 in the Tibet Autonomous Region, with a design speed of 20 km/h, a subgrade width of 7.5 m, and pavement width 6.5 m. The road pile numbers are K4+812–K5+107 and K7+501–K7+865, respectively. From May to September 2025, municipal solid waste incinerator bottom ash aggregates were applied in these sections for engineering purposes. The bottom ash was mainly sourced from Lhasa, approximately 70 km away. The two trial stretches did not adopt the same OAC; instead, the asphalt–aggregate ratio was separately adjusted according to the bottom ash particle size fraction and the Marshall design results. The pavement structures of the test sections are presented in Table 6 and Table 7.

### 4.2. Performance Testing of the Test Sections

The S303 test sections are located in Dangxiong, Lhasa, China, within the alpine pastoral area of Northern Tibet, at an average elevation of 4300 m. The local climate and traffic conditions are characterized by large diurnal temperature variations, intense ultraviolet radiation, repeated freeze–thaw cycles of snow and ice, and high-frequency heavy-load traffic [42]. Therefore, the test sections were primarily evaluated through core sampling, low-temperature crack resistance, moisture stability, and fatigue performance tests.

(1) Core sampling results

**Figure 8:**
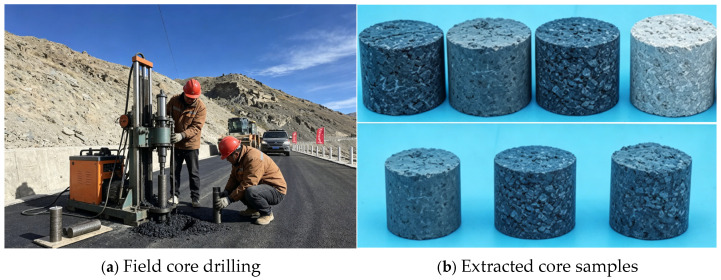
Core sampling of the test sections.

To assess the overall condition of the pavement structure, particularly the integrity of the base course, a total of seven core samples were extracted from Test Sections 1 and 2 in October 2025, approximately one month after paving completion and early traffic opening. Field core drilling is shown in Figure 8a, and the extracted core samples are shown in Figure 8b. The loose material visible near the drilling machine in Figure 8a was not uncompacted pavement mixture, but rather drilling residue and water slurry produced during coring. The basic conditions of the samples are presented in Table 8.

The core sampling results and pavement overall condition investigation indicate that the surface and base courses of Test Sections 1 and 2 maintained good integrity during the early field verification period, and the preliminary service performance of the asphalt pavement using MSWI bottom ash remained satisfactory.

(2) Pavement performance testing of the test sections

**Figure 9:**
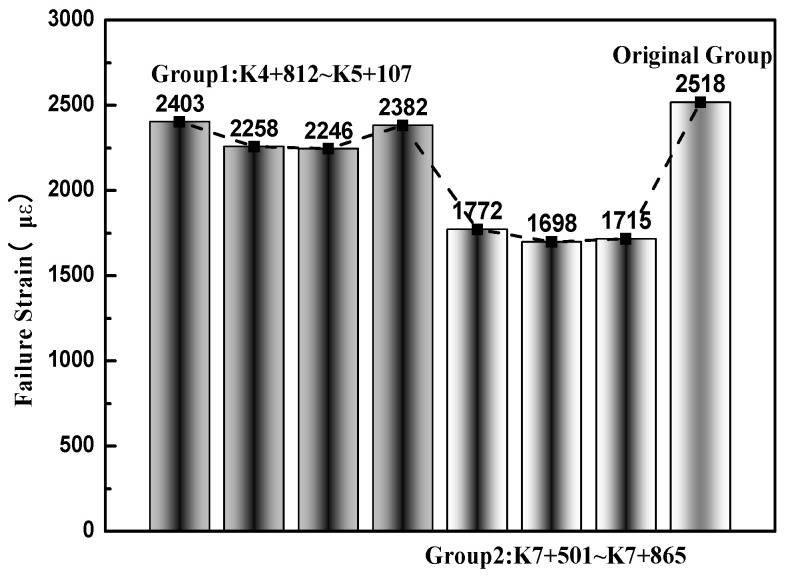
Low-temperature crack resistance test results.

**Figure 10:**
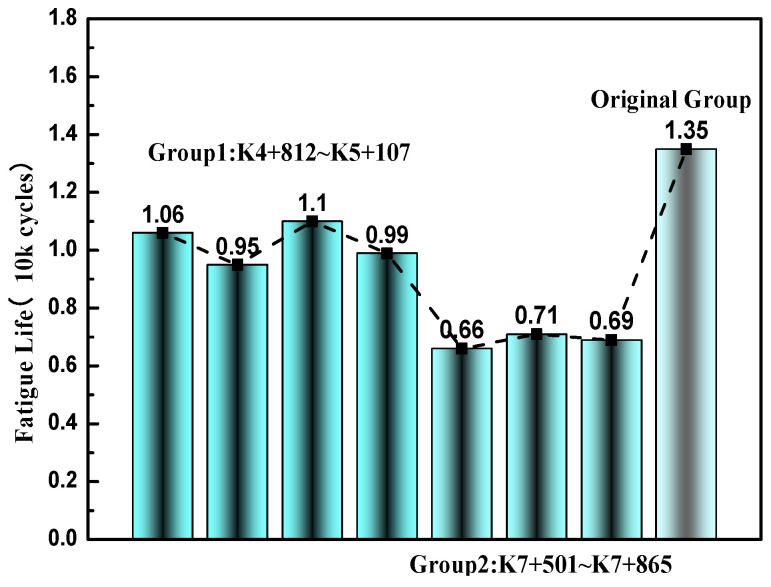
Fatigue performance test results.

**Figure 11:**
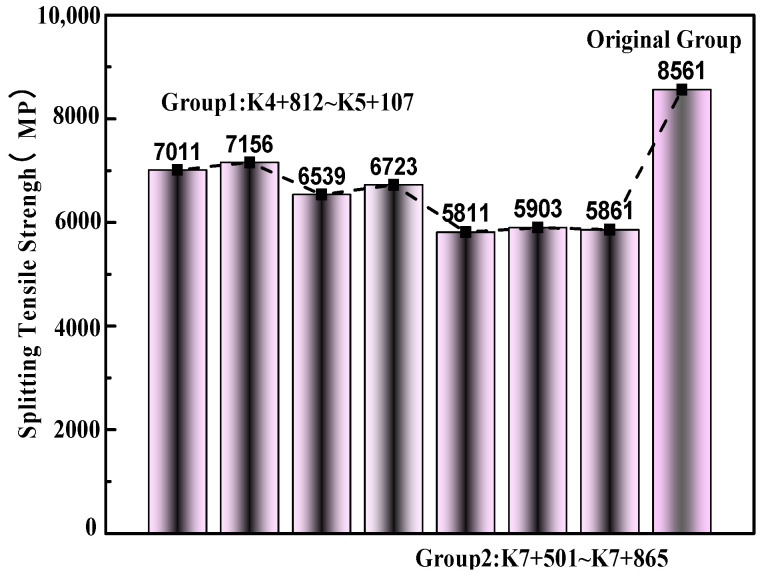
Water stability test results.

According to the specification [43], laboratory tests were conducted on a total of eight experimental groups, comprising the seven core samples extracted from the field (see Table 8) and the original pavement. The bending test was performed at a test temperature of −10 °C with a loading rate of 50 mm/min, using specimens cut to dimensions of 250 mm × 30 mm × 35 mm. This beam size follows the small-beam bending method specified in JTG E20-2011 and was adopted because the field core thickness was limited by the 5 cm AC-20 surface course; specimens with visible aggregate discontinuity or cutting damage were discarded before testing. Fatigue resistance was evaluated by a four-point bending fatigue test at a test temperature of 15 °C and a loading frequency of 10 Hz. Water stability was assessed using the freeze–thaw splitting test, in which the splitting strength of the mixture before and after freeze–thaw cycles was measured to calculate the freeze–thaw splitting strength ratio, thereby evaluating the mixture’s resistance to moisture damage. The test results are shown in Figure 9, Figure 10 and Figure 11.

As shown in Figure 9, Figure 10 and Figure 11, the addition of 25% slag to the asphalt mixture resulted in lower performance indicators for Test Sections 1 and 2 compared to the original pavement, indicating that slag degrades the performance of asphalt mixtures in practical engineering applications. In terms of numerical values, the performance indicators of Test Section 1 exhibited a relatively small reduction, generally within 10%, which is considered acceptable. However, the indicators of Test Section 2 decreased by more than 30%, with the fatigue resistance showing a reduction of up to 50%. This field comparison further supports the laboratory finding that a small particle size fraction should be preferentially selected in alpine and high-altitude regions. Therefore, the mixing ratio is recommended to be below 25% for field application, and bottom ash with a large particle size range is not recommended for direct blending in the asphalt surface course.

## 5. Conclusions

This study evaluated the feasibility of using MSWI bottom ash as an aggregate replacement in SBS-modified AC-20 asphalt mixtures through laboratory tests and field trial sections. The main conclusions are as follows:

(1) MSWI bottom ash causes different degrees of degradation in the pavement performance of modified asphalt mixtures. At 30% dosage, the dynamic stability of Groups I, II and III decreased by 37.5%, 49.3% and 27.5%, respectively, while fatigue life decreased by 48.1%, 60.3% and 31.3%, respectively. The deterioration is mainly related to the high crushing value, high water absorption, relatively poor aggregate strength and weak adhesion between bottom ash and asphalt.
(2) The particle size fraction of MSWI bottom ash is a key factor controlling mixture performance. The 2.36–4.75 mm fraction produced the smallest performance attenuation, followed by the 2.36–9.5 mm fraction, whereas the 9.5–16 mm fraction caused the most obvious deterioration. This indicates that small-sized bottom ash is more suitable as a partial alternative aggregate in asphalt mixtures.
(3) Considering comprehensive pavement performance and resource utilization efficiency, the recommended replacement dosage is not more than 30% for the 2.36–4.75 mm fraction and not more than 22.5% for the 2.36–9.5 mm fraction. Direct replacement using the large 9.5–16 mm fraction is not recommended for the surface course asphalt mixture, especially under alpine, freeze–thaw and heavy-load conditions.
(4) The field trial sections preliminarily verified the engineering feasibility of bottom ash asphalt mixtures, but the performance reduction in the large-particle bottom ash section was more significant than that of the small-particle bottom ash section. Therefore, future engineering applications should combine particle size control, OAC adjustment and continuous field monitoring.
(5) The study still has limitations. The bottom ash was obtained from a single source, and the field monitoring period was relatively short. In addition, radon concentration and gamma radiation emissions from the bottom ash were not measured in this study. Although the tested bottom ash was used only after sorting and aggregate performance evaluation, environmental radiation and long-term leaching behavior should be included in subsequent studies before wider application.

## Figures and Tables

**Table 1:** Contents of main chemical compositions of MSWI slag (%).

Main Components	SiO_2_	Fe_2_O_3_	Al_2_O_3_	CaO	Na_2_O	Others
Contents (%)	36.6	10.5	6.8	30.5	8.9	6.7

**Table 2:** Performance indicators of aggregates and mineral fillers.

Parameters	Basalt Coarse Aggregate	MSWI Slag (Beyond the Permissible Limit of F40 Sieve)	Limestone Fine Aggregate	Mineral Filler	Test Basis
Crushing Value (%)	7.9	28.9	/	/	JTG E42-2005 [20] T0316
High-Temperature Crushing Value (%)	8.6	31.1	/	/	JTG E42-2005 [20] T0316
Los Angeles Abrasion Loss (%)	8.2	30.6	/	/	JTG E42-2005 [20] T0317
Flaky and Elongated Particle Content (%)	5.4	12.5	/	/	JTG E42-2005 [20] T0312
Water Absorption (%)	1.68	4.8	/	/	JTG E42-2005 [20] T0304
Content of Particles Smaller than 0.075 mm by Water Washing Method (%)	0.41	4.86	/	/	JTG E42-2005 [20] T0310
Sand Ratio (%)	/	/	78.21	/	JTG E42-2005 [20] T0334
Apparent Density (g/cm^3^)	2.958	2.467	2.980	2.685	JTG E42-2005 [20] T0304/T0328
Bulk Specific Gravity (g/cm^3^)	2.876	2.388	2.920	2.685	JTG E42-2005 [20] T0304/T0328

**Table 3:** Performance indicators of SBS-modified asphalt.

Index	Penetration (0.01 mm)	5 °C Ductility (cm)	Softening Point (°C)	135 °C Viscosity (Pa·s)	Density (g/cm^3^)	RTFOT After Aging		
						Penetration Ratio (%)	Residual Ductility (cm)	Mass Change (%)
Measured Value	57.5	35.1	72.4	5.4	1.028	85.5	26.1	0.24
Requirement Value	40–60	≥20	≥60	≤3	/	≥65	≥15	±1
Test basis	JTG E20-2011 T0604	JTG E20-2011 T0605	JTG E20-2011 T0606	JTG E20-2011 T0625	JTG E20-2011 T0603	JTG E20-2011 T0610	JTG E20-2011 T0610	JTG E20-2011 T0610

**Table 4:** Sieve passing rates of AC-20 asphalt mixture (%).

Particle Size (mm)	26.5	19	16	13.2	9.5	4.75	2.36	1.18	0.6	0.3	0.15	0.075
Percent Passing (%)	100	96.5	85.2	71.1	62.5	39.1	28.9	17.2	12.3	8.6	5.2	4.0

**Table 5:** Characteristics of the experimental mixture series.

Series	Particle Size of MSWI Bottom Ash (mm)	Share of Replacement Interval in Total Aggregate (%)	Bottom Ash Dosage (%)	OAC Design
Control	-	-	0	Determined by Marshall test
Group I	2.36–9.5	33.6	7.5, 15, 22.5, 30	Adjusted with dosage according to Marshall test
Group II	9.5–16	34.0	7.5, 15, 22.5, 30	Adjusted with dosage according to Marshall test
Group III	2.36–4.75	10.2	7.5, 15, 22.5, 30	Adjusted with dosage according to Marshall test

**Table 6:** Pavement structure of Test Section No. 1 (S303 K4+812~K5+107).

Test Section Pavement Structure	Original Pavement Structure	Remarks
AC-20 SBS-modified asphalt concrete 5 cm (blended with 2.36–4.75 mm furnace slag at a blending ratio of 25%)	AC-20 SBS-modified asphalt concrete 5 cm	Surface course
Synchronous chip seal	Synchronous chip seal	Seal coat
Cationic emulsified asphalt type PC-2, thickness 6 mm	Cationic emulsified asphalt type PC-2, thickness 6 mm	Prime coat
20 cm cement-stabilized crushed stone	20 cm cement-stabilized crushed stone	Base course
20 cm graded crushed stone	20 cm graded crushed stone	Subbase course

**Table 7:** Pavement structure of Test Section No. 2 (S303 K7+501~K7+865).

Test Section Pavement Structure	Original Pavement Structure	Remarks
AC-20 SBS-modified asphalt concrete 5 cm (blended with 9.5–16 mm furnace slag at a blending ratio of 25%)	AC-20 SBS-modified asphalt concrete 5 cm	Surface course
Synchronous chip seal	Synchronous chip seal	Seal coat
Cationic emulsified asphalt type PC-2, thickness 6 mm	Cationic emulsified asphalt type PC-2, thickness 6 mm	Prime coat
20 cm cement-stabilized crushed stone	20 cm cement-stabilized crushed stone	Base course
20 cm graded crushed stone	20 cm graded crushed stone	Subbase course

**Table 8:** Core sampling results of the S303 test sections.

	Road Pile Numbers	Core Sample No.	Pavement Surface Condition	Core Description
Test Section 1 (K4+812~K5+107)	K4+900	1	Good	Surface and base courses intact
	K4+940	2	Good	Surface and base courses intact
	K4+980	3	Good	Surface and base courses intact
	K5+020	4	Good	Surface and base courses intact
Test Section 2 (K7+501~K7+865)	K7+600	5	Good	Surface and base courses intact
	K7+700	6	Good	Surface and base courses intact
	K7+800	7	Good	Surface and base courses intact

## Data Availability

The original contributions presented in this study are included in the article. Further inquiries can be directed to the corresponding author.

## References

[1] Chen L., Wen S., Luo J., Wang Y. (2025). Combustion characteristics and kinetic analysis of co-combustion of home decoration wood waste and municipal solid waste. Clean Coal Technol..

[2] Zhang J., Zheng M., Zheng F., Yang J., Zheng Y. (2025). Characterization of domestic waste leachate generation, treatment methods, and development trends in China: A comprehensive review. Chin. J. Environ. Eng..

[3] Deng T., Ma Z., Liu S., Gao Q., Gao W., Li H., Xie L. (2024). Characteristics of CH_4_ and VOCs emissions from municipal solid waste landfills in China from 2002 to 2022. China Environ. Sci..

[4] Dias J.F., Picado-Santos L.G., Capitão S.D. (2014). Mechanical performance of dry process fine crumb rubber asphalt mixtures placed on the Portuguese road network. Constr. Build. Mater..

[5] Zhu J., Ma T., Fang Z. (2020). Characterization of Agglomeration of Reclaimed Asphalt Pavement for Cold Recycling. Constr. Build. Mater..

[6] Peng X., Tang S., Tang Y., Ma X., Bai X., Song X., Fan S., Cao B. (2025). Environmental impact and optimization of the greenhouse gas emission reduction of the whole chain of waste incineration based on life cycle assessment: A case study of Shenzhen. Environ. Pollut. Control..

[7] Liu S., Li C., Xu L. (2021). Research progress on treatment technology of MSWI fly ash. Appl. Chem. Ind..

[8] Zhang H., Gong M., Gao D., Yang T., Huang Y. (2020). Comparative analysis of mechanical behavior of composite modified. asphalt mixture based on PG technology. Constr. Build. Mater..

[9] Zhang Y., Zhao X. (2026). Disulfide-Crosslinked Polyurethane-Modified Asphalt: Balancing Fatigue Resistance and Healing Through Dynamic Covalent Networks. Polymers.

[10] Yu J., Chen F., Deng W., Ma Y., Yu H. (2020). Design and performance of high-toughness ultra-thin friction course in south China. Constr. Build. Mater..

[11] Vasconcellos A.T., Matos P.R., Casagrande C.A., Ribeiro A.V.S., Prudêncio L.R. (2021). Evaluating the variability of the modulus of elasticity of concrete through the use of different types and batches of aggregate. Matéria.

[12] Oliveira F.A., Casagrande C.A., Marinho É.P., Jochem L.F., Nóbrega A.C.V. (2022). Blasted copper slag as artificial fines in ecofriendly concrete. Matéria.

[13] Fang K., Zhou Q., Wang J., Feng Z., Wu X. (2025). Research progress on the resource utilization of municipal solid waste incineration bottom ash in concrete. Environ. Sanit. Eng..

[14] Xu L., Nie Y., Shen L. (2024). Analysis of the current utilizing status on the resource utilization of municipal solid waste incineration slag. Ecol. Resour..

[15] Zhou S., Zeng C., Feng B., Xie G., Li Z., Liu G., Wang Z., Liu J. (2023). Research on the application of municipal solid waste incineration bottom ash in road base. Highway.

[16] Dong Z. (2024). Experimental Study on Road Performance of Asphalt Mixture Mixed with Recycled Aggregate and Slag Aggregate. Master’s Thesis.

[17] Zhang H. (2025). Research on the influence of titanium-containing blast furnace slag on the structure and hydration characteristics of belite cement. China Nonferrous Metall..

[18] Zhang T., Zhao Z. (2014). Reutilization of municipal solid waste in-cinerator bottom ash as concrete aggregates. Environ. Pollut. Control..

[19] Ding H., Gong H., Cong L., Hou Y. (2024). Investigating influence of hard segment content on rheological behavior of thermosetting PU modified asphalt. Constr. Build. Mater..

[20] (2005). Standard Test Methods of Aggregate for Highway Engineering.

[21] (2017). Technical Specifications for Construction of Highway Asphalt Pavements..

[22] (2011). Standard Test Methods of Bitumen and Bituminous Mixtures for Highway Engineering.

[23] He Y., Xing C., Hong B., Tan Q.W., Wang D.W., Oeser M. (2022). Influence of Polished Stone Values of Coarse and Fine Aggregates on Long-Term Skid Resistance of Asphalt Pavements. China J. Highw. Transp..

[24] Liu Z., Sha A., Jiang W. (2019). Research Progress of Salt-Storing Asphalt Pavement: Chloride Materials, Mixtures, Performance and Evaluation. China J. Highw. Transp..

[25] Wang Y., Yi L., Zheng W., Gu M., Wang J. (2026). Compaction characteristics and high-compaction paving study of AC-13 steel slag asphalt concrete. J. China Foreign Highw..

[26] Zhang J., Li G., Ma Y., Jiang S., Xing H., Zhang F. (2016). Study on preparation and properties of slag concrete. Concrete.

[27] Song P. (2026). Research on Construction Quality Control of Large Void Drainage Asphalt Pavement of Expressway. Constr. Des. Proj..

[28] Zhang Q., Song Y., Wu S., Jiang Q., Cheng S., Lin K. (2026). Study on Pavement Performance of Steel Slag-Epoxy Asphalt Ultra-Thin Wearing Course. J. China Foreign Highw..

[29] He L., Zhan C., Lv S., Grenfeell J., Gao J., Kowalski K., Valentin J., Xie J., Rek L., Ling T. (2020). Application Status of Steel Slag Asphalt Mixture. J. Traffic Transp. Eng..

[30] Gao Z., Shen A., Zhai C., Guo Y., Yu P. (2018). Determination of volumetric parameters and impacting mechanism of water stability for steel slag asphalt mixture. J. Traffic Transp. Eng..

[31] Hu J., Zhao W., Wen W., Guo Y., Yu P. (2025). Investigation of Long-term Moisture-induced Damage in Asphalt Mixtures Fully Replaced with Steel Slag Aggregate. China J. Highw. Transp..

[32] Kong L., Wang Z., Su S., Yue J., Luo W., Zhou S., Ai C. (2024). Exploring the interplay between thermo-oxidative degradation asphalt aging in thermoplastic polyurethane-modified asphalt: Mechanisms, properties, and performance evolution. Constr. Build. Mater..

[33] Sun L., Xu X., Gu X., Hu D., Zhou Z. (2025). Enhanced antioxidant activity of lignin for improved asphalt binder performance and aging resistance in sustainable road construction. Constr. Build. Mater..

[34] Hu M., Sun D., Sun G., Yu F., Sun Y., Zhou C. (2023). Insights into long-term environmental oxidation of recycled high-viscosity modified asphalt toward durable and sustainable pavement infrastructure. Constr. Build. Mater..

[35] Ma R., Li Y., Cheng P., Chen X., Cheng A. (2024). Low-temperature cracking and improvement methods for asphalt pavement in cold regions: A review. Buildings.

[36] Wu S., Cui P., Xie J., Liu Q., Pang L. (2021). Research status of expansion inhibition methods for steel slag aggregate and volume stability of mixtures. China J. Highw. Transp..

[37] Zhou M., Liu S., Wu C., Liu J., Zhang H., Zhang S., Li Q. (2024). Gradation design and performance comparative study of ultra-thin wearing course based on waterborne epoxy emulsified asphalt. Mater. Rep..

[38] Sun Y., Li L. (2017). Effect of Municipal Solid Waste Incineration Bottom Ash Powder on Properties of Asphalt Mixture. J. Tongji Univ..

[39] Zhang R., Zhang T. (2010). Characteristics of waste incineration bottom ash and its influence on compression strength of concrete. Highway.

[40] Joumblat R., Al Basiouni Al Masri Z., Elkordi A. (2023). Dynamic Modulus and Phase Angle of Asphalt Concrete Mixtures Containing Municipal Solid Waste Incinerated Fly Ash as Mineral Filler Substitution. Int. J. Pavement Res. Technol..

[41] Bruneau L., Tisse S., Michon L., Cardinael P. (2023). Investigation of Oxidation Homogeneity in Asphalt Puck after Simulation of Long-Term Aging (Pressure Aging Vessel). Materials.

[42] Wang W., Wang L., Xiong H., Luo R. (2019). A review and perspective for research on moisture damage in asphalt pavement in-duced by dynamic pore water pressure. Constr. Build. Mater..

[43] Wang W., Wang L., Yan G., Zhou B. (2020). Evaluation on moisture sensitivity of asphalt mixture induced by dynamic pore water pressure. Int. J. Pavement Res. Technol..

